# Prevalence of epilepsy in the onchocerciasis endemic middle belt of Ghana after 27 years of mass drug administration with ivermectin

**DOI:** 10.1186/s40249-023-01117-9

**Published:** 2023-08-17

**Authors:** Kenneth Bentum Otabil, Blessing Ankrah, Emmanuel John Bart-Plange, Emmanuel Sam Donkoh, Fiona Amoabil Avarikame, Fredrick Obeng Ofori-Appiah, Theophilus Nti Babae, Prince-Charles Kudzordzi, Vera Achiaa Darko, Joseph Ameyaw, Joseph Gyekye Bamfo, Raji Abdul Sakibu, Daniel Antwi-Berko, Joseph Nelson Siewe Fodjo, María-Gloria Basáñez, Henk D. F. H. Schallig, Robert Colebunders

**Affiliations:** 1 NeTroDis Research Group, Centre for Research in Applied Biology, School of Sciences, University of Energy and Natural Resources, Bono Region, Sunyani, Ghana; 2 Department of Biological Science, School of Sciences, University of Energy and Natural Resources, Bono Region, Sunyani, Ghana; 3 Global Health Institute, University of Antwerp, Antwerp, Belgium; 4 Department of Medical Laboratory Science, School of Sciences, University of Energy and Natural Resources, Bono Region, Sunyani, Ghana; 5 STU Clinic, Sunyani Technical University, Bono Region, Sunyani, Ghana; 6 Happy Family Hospital, Bono East Region, Nkoranza, Ghana; 7 Tain District Hospital, Bono East Region, Nsawkaw, Ghana; 8 Department of Infectious Disease Epidemiology, MRC Centre for Global Infectious Disease Analysis (MRC GIDA), and London Centre for Neglected Tropical Disease Research, School of Public Health, Imperial College London, London, UK; 9 Department of Medical Microbiology, Experimental Parasitology Unit, Amsterdam University Medical Centres, Academic Medical Centre at the University of Amsterdam, Amsterdam, The Netherlands

**Keywords:** *Onchocerca volvulus*, Microfilaria, Onchocerciasis-associated epilepsy, Seizure, Ivermectin, Mass drug administration, Coverage

## Abstract

**Background:**

In onchocerciasis-endemic areas with high ongoing *Onchocerca volvulus* transmission, a high prevalence of epilepsy has been reported. This study aimed to determine the prevalence and clinical characteristics of epilepsy in the Bono Region of Ghana following 27 years of implementation of ivermectin mass drug administration (MDA).

**Methods:**

Between October 2020 and August 2021, cross-sectional surveys were conducted in nine communities in the Tain District and Wenchi Municipality of the Bono Region of Ghana. In the first stage, a random door-to-door approach was used to screen the population for epilepsy using a pre-tested questionnaire. Persons suspected of having epilepsy were invited for a second-stage neurological examination for case verification. Community *O. volvulus* microfilarial infection status and Ov16 seropositivity were also determined. Ninety-five confidence intervals (95% *CI*) for prevalence values were calculated using the Wilson Score Interval.

**Results:**

Of the 971 participants, 500 (51.5%) were females, and the median age (interquartile range) was 26 (15‒43) years. Fourteen participants (1.4%, 95% *CI*: 1.0‒2.0) were diagnosed as having epilepsy with generalized seizures being the most frequent seizure type (85.7%, 12/14). The overall microfilarial prevalence of *O. volvulus* was 10.3% (November 2020) and 9.9% (August 2021); the Ov16 seroprevalence was 22.2% (June 2021). Only 63.2% took ivermectin in the last round of MDA distribution in March 2021.

**Conclusions:**

The 1.4% prevalence of epilepsy in the Bono region is similar to the median epilepsy prevalence in sub-Saharan Africa. However, the persistent microfilarial prevalence and low ivermectin study coverage call for the Ghana Onchocerciasis Elimination Programme to step up its efforts to ensure that the gains achieved are consolidated and improved to achieve the elimination of onchocerciasis by 2030.

**Graphical Abstract:**

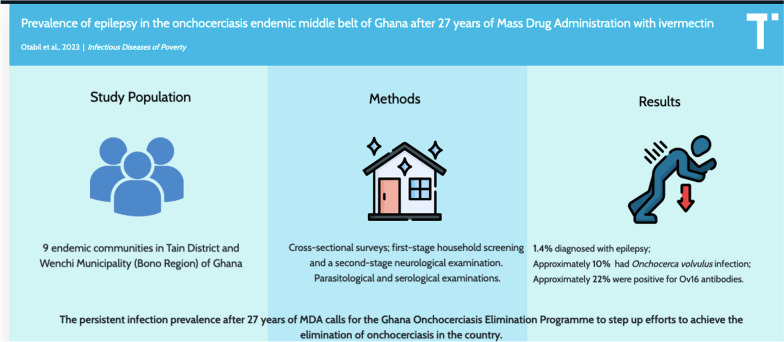

**Supplementary Information:**

The online version contains supplementary material available at 10.1186/s40249-023-01117-9.

## Background

Onchocerciasis, a severe neglected tropical disease (NTD) still plagues about 21 million individuals from the poorest of the poor populations, mostly in sub-Saharan Africa (SSA), with small endemic foci in Brazil, Venezuela, and Yemen [1]. The disease is caused by infection with the filarial nematode *Onchocerca volvulus*, and is transmitted by blackflies of the genus *Simulium*, which breed in fast-flowing, well-oxygenated rivers [2]. Onchocerciasis has huge socio-economic and psychosocial implications for the unfortunate sufferers and their families due to its clinical manifestations, including intense chronic dermatitis, subcutaneous nodules, skin atrophy, visual impairment and blindness, among others [3]. About 500,000 cases of severe visual impairment and 270,000 cases of blindness were officially attributed to onchocerciasis prior to the inception of regional control initiatives, but these figures are thought to have been underestimates of the true burden of the disease [4], with other (baseline) estimates indicating figures nearly twice as high, with approximately 900,000 suffering from visual impairment and 400,000 people blind due to onchocerciasis [5].

Aside from the well-known dermatologic and ophthalmic pathologies associated with onchocerciasis, there is a growing body of evidence for the association between onchocerciasis and the development of epilepsy, known as onchocerciasis-associated epilepsy (OAE) [6]. Though recognition of this association dates back to 1938, an etiological link is still pending universal acceptance [7]. This notwithstanding, several epidemiological studies have demonstrated a high prevalence of epilepsy, especially in areas that are meso- and hyperendemic for onchocerciasis where the transmission of the parasite is very intense and/or not well-controlled due to the sub-optimal implementation of mass drug administration (MDA) with ivermectin programmes [7–11].

Ghana is one of the countries where initial community trials of ivermectin were performed in 1987–1991 [12], and was among the first countries to start an MDA programme with ivermectin in 1995, via mobile teams [13]. Fifteen out of the sixteen regions of Ghana are endemic for onchocerciasis. The Community Directed Treatment with Ivermectin (CDTI) strategy was implemented in all of them, with all endemic communities receiving treatment annually after 1998 [13]. Following the cessation of the Onchocerciasis Control Programme in West Africa (OCP) in 2002, some regions, classified as Special Intervention Zones (SIZ; areas where microfilarial prevalence remained above 50%, and identified as of concern by the OCP), received further interventions beyond 2002. In Ghana, SIZs included an area in the Pru River basin, where CDTI continued yearly till 2012 [14].

Despite the implementation of CDTI in the late 1990s, inadequate financial support coupled with management challenges led to the erratic distribution of ivermectin with poor therapeutic and geographical coverage for most of the treatment areas [13]. The inception of the lymphatic filariasis (LF) elimination programme in 2000 led to the distribution of ivermectin and albendazole to many (co-endemic) communities in Ghana [15]. From 2004, drug distribution as part of the LF elimination programme was combined with the onchocerciasis control programme, resulting in substantial improvements in geographical and therapeutic coverage. There was a gradual increase in the number of people treated and epidemiological coverage. In 2016, over 4 million individuals in endemic communities were treated. The therapeutic coverage increased from 58.5% in 1997 to 83.8% in 2016. After 2006, the coverage was consistently above 65% [13].

In the initial stages of CDTI, up-to-date mapping data were not available for the country, so the programme relied on historical data compiled from regional and district health teams, as well as community surveys, to guide the treatment for onchocerciasis. In 2008, Rapid Epidemiological Mapping of Onchocerciasis (REMO) [16] was undertaken in Ghana, with the support of the African Programme for Onchocerciasis Control (APOC), to rapidly re-map and identify onchocerciasis-endemic communities (based on onchocercal nodule prevalence). Endemicity maps were developed for the preparation of a national plan for onchocerciasis control, and for a re-launch of ivermectin MDA in the newly identified communities. High-risk communities were selected (communities in the immediate vicinity of major potential vector breeding sites) [13]. Blackflies are known to have a typical flight range of 20 km around their breeding sites (although, wind-aided, they may fly distances of at least 400 km [17, 18]). Communities were, therefore, selected for treatment along the identified rivers within the 20 km flight range of the blackflies [13]. In 2009, biannual (6-monthly) treatment with ivermectin was instituted in 55% of districts. The number of communities treated gradually increased from 2009 [19].

The epidemiology and socio-economic burden of the dermatological and ophthalmological pathologies of onchocerciasis, as well as the impact of ivermectin MDA on such pathologies, have been assessed in several studies. However, to date, only one study conducted in 2010‒2011 [20] investigated the prevalence of epilepsy in an onchocerciasis-endemic area in Ghana. The prevalence of a history of convulsions in the demographic surveillance site of Kintampo, in the middle belt of Ghana, was 2.9% (95% confidence intervals (95% *CI*): 2.8‒3.0%) and persons with epilepsy were twice as likely to present *O. volvulus* IgG4 antibodies compared to controls (odds ratio = 2.32; 95% *CI*: 1.12‒4.78) [20].

Given the long history of onchocerciasis interventions in Ghana, the aim of this study was to determine the prevalence and clinical characteristics of epilepsy in onchocerciasis-endemic communities in the Bono Region of Ghana, where ivermectin MDA has been implemented for the past 27 years.

## Methods

### Study area and history of control in the study villages

**Fig. 1:**
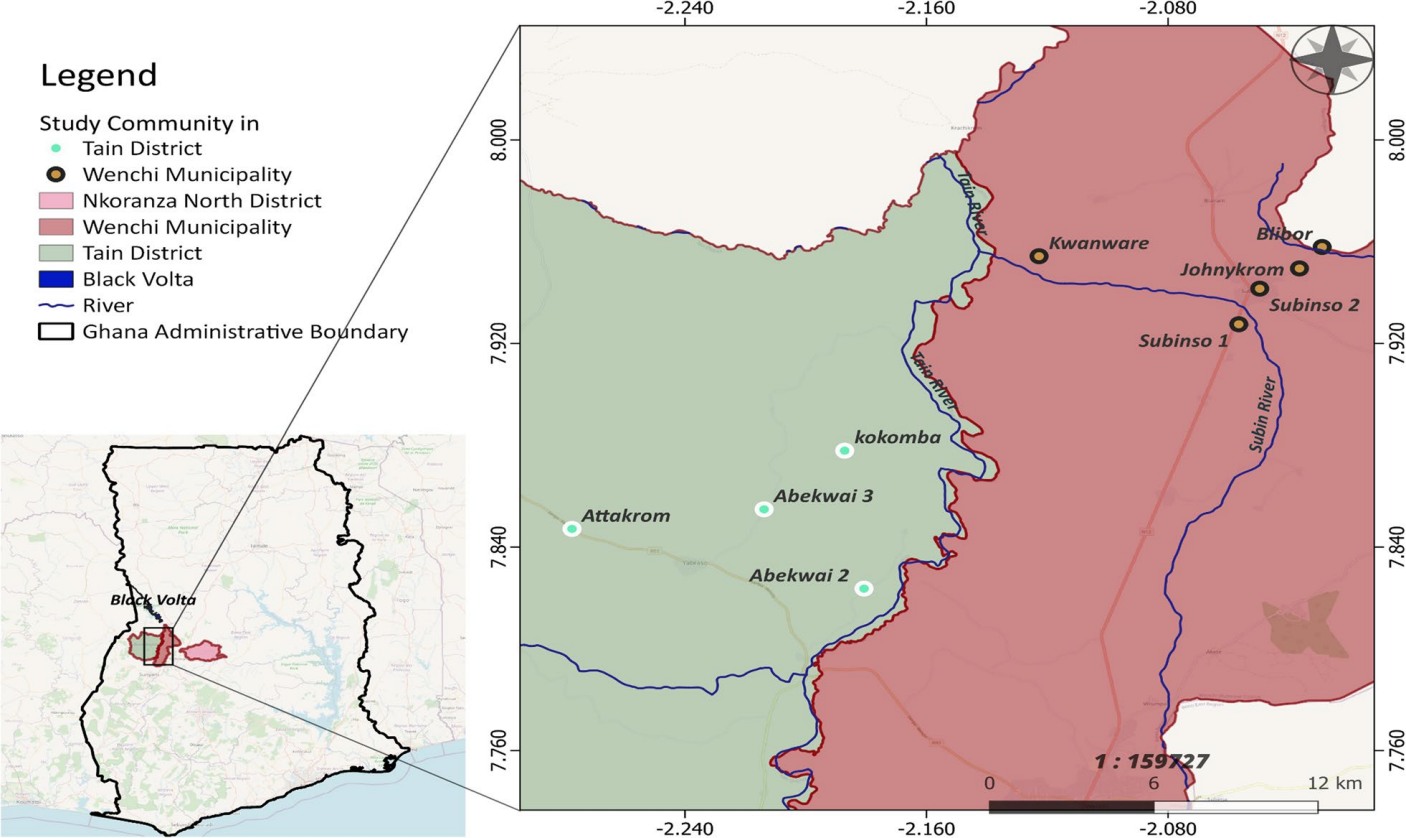
Map of Ghana showing the location of the study communities within the Tain District and Wenchi Municipality (Bono Region)

This study was performed from October 2020 to August 2021 in nine rural onchocerciasis-endemic communities in the Tain District and Wenchi Municipality of the Bono Region of Ghana (Fig. 1). The Tain District has a total area of 1829.85 square kilometres and a population size of 88,104 consisting of 50.6% females and 49.4% males [21]. The Wenchi Municipality has a total area of 1296.60 square kilometres and a population of 89,739, with 50.9% females and 49.1% males [22]. The selected study communities were Abekwai 2, Abekwai 3, Attakrom, and Kokomba in the Tain District, and Blibor, Johnykrom, Subinso 1, Subinso 2, and Kwanware in the Wenchi Municipality (Fig. 1). This map indicates that the study villages consist of two clusters, one cluster of four villages in Tain District to the west of the main Tain river, and one cluster of five villages along the Subin River in Wenchi Municipality (the maximum cluster diameter is 11 and 8 km, respectively). It is important to differentiate these two clusters because their vector control history is different. The section of the main Tain river from the village of Tainso till the junction with the Black Volta started vector control in 1976 as part of Phase II of the OCP. The Subin river was part of the South-Eastern Extension of the OCP that became operational in 1988. The Subin river was under vector control from 1992 onwards but vector control may have started a few years earlier. Vector control ended in both the Tain and Subin rivers in 1996. The baseline *O. volvulus* (crude) microfilarial prevalence for Kwanware (in the Wenchi cluster) in 1989 was 48.1% (95% *CI*: 41.5–54.8%) and the community microfilarial load (CMFL) was 7.26 microfilariae per skin snip (mf/ss), indicating mesoendemicity according to OCP data. In a survey conducted by the OCP in the year 2000, these values had decreased to 15.6% (95% *CI*: 10.0–23.6%) and 0.33 mf/ss, respectively, and in another survey conducted in 2012 (supported by APOC Trust Fund), the mf prevalence was 5.6% (95% *CI*: 2.2–13.6%). All the other study communities in the Wenchi cluster lie within 8 km of Kwanware and are located within the Subin river basin. For the Tain cluster, there are no data on pre-control microfilarial prevalence for our specific study villages. However, there is one nearby OCP village, Tainso, for which pre-control (crude) microfilarial prevalence is available for 1980 (40.7%, 95% *CI*: 35.4–46.2%) and which is situated within 4–10 km from each study village in the Tain cluster, also indicating mesoendemicity. The villages in both clusters have received MDA by mobile teams since 1994–1995, and CDTI since 1998. They have, therefore, had ivermectin MDA programmes for the past 27 years. Since 2009–2010, the onchocerciasis control programme in our study area switched from annual to biannual ivermectin MDA in Tain and subsequently in Wenchi [13] (Additional file 3: Table S1).

### Ethical clearance

The study was approved by the Committee for Human Research and Ethics of the University of Energy and Natural Resources in Sunyani, Ghana, West Africa (Approval number: CHRE/AP/08/021). In each of the study communities, a meeting with the community head and key opinion leaders was held during which the study was discussed and consent was obtained. In each household, a written, signed/thumb printed consent of the household heads, as well as each participant and/or their parent/guardian, was obtained. In addition to the written consent of their parents/guardians, children between 10 and 17 years were asked to provide assent to the consent form.

### Study design

This was a population-based cross-sectional study, using a door-to-door approach, the gold standard for neuro-epidemiological surveys to identify epilepsy cases in Low- and middle-income countries (LMICs) [23]. For small communities (≤ 40 households), all households were included; for larger communities (> 40 households), households were randomly selected. In the first stage, members of selected households were screened by trained research assistants to identify persons suspected of having epilepsy using a pretested questionnaire (Additional file 1). The questionnaire consisted of five epilepsy screening questions adapted from a previously validated questionnaire in Mauritania [24]. If a person answered ‘Yes’ to any of the five questions on epilepsy, then they were considered suspected epilepsy cases and were referred to the medical team for the second stage neurological examinations. Each household member was also asked whether they had taken ivermectin during the previous distribution round. The household screening tool also collected data about the duration of residence in the community, the main income-generating activity of the family, exposure to pigs (a risk factor for *Taenia solium* taeniasis/cysticercosis, as neurocysticercosis is also associated with epilepsy), and whether household members had died of epilepsy in the past. In the second stage, suspected cases of epilepsy identified during the household screening surveys were invited for clinical examination by a medical team made up of a medical doctor (JA) and a physician assistant (JGB) who either confirmed the diagnosis of epilepsy or suggested an alternative diagnosis. The diagnosis by the field medical team was also confirmed independently by a second clinician (VAD). The medical team was trained to diagnose epilepsy as per the International League Against Epilepsy (ILAE) guidelines: having experienced at least two previous seizures, unprovoked and without fever, with a minimal time difference of 24 h between the two events [25]. The neurological questionnaire (Additional file 2) included questions about the onset of epilepsy, symptoms, clinical characteristics, types of seizures, psychomotor development, etc. Participants were also asked about the occurrence of severe diseases prior to developing epilepsy, whether they were known to have epilepsy, how they were treated for epilepsy, and to recall their pattern of ivermectin intake over the past years as well as during the most recent treatment round of March 2021.

### Data collection and management

During the household screening stage, data gathered during the screening stage were collected on the Kobocollect app [26] using Android tablets. To clinically confirm suspected epilepsy cases, the medical team used a second questionnaire also deployed on KoboCollect. Unique codes were assigned to each suspected case of epilepsy to link their parasitological and serological data.

### Parasitological diagnosis of onchocerciasis

During 2020, due to the coronavirus disease 2019 (COVID-19) pandemic, CDTI was not delivered. Therefore, in November 2020 we collected parasitological data one year after the last treatment round (in October 2019). During 2021, there were two treatment rounds, the first in March; hence the data collected in August correspond to five months after treatment. (Another treatment round took place immediately after our data collection in August 2021.) Both in 2020 and 2021, all participants were invited to a central gathering point in the community to participate in the studies aimed to determine the prevalence of *O. volvulus* microfilarial infection*.* All who consented to participate were skin-snipped following established protocols [27]. Briefly, a piece of cotton wool soaked in methylated spirit was used to clean both sides of the iliac crests of each person. Blood-free skin snips (approx. 2 mg) were taken from each individual using a sterilized 2-mm Holth corneoscleral punch (E1802, Holth Storz, Germany). Biopsies were weighed and then separately incubated in physiological saline in 96-well microtitration plates (Caplugs Evergreen 222-8052-R1K, USA) for 24 h. Microscopic examination to identify and detect *O. volvulus* microfilariae was performed.

### Testing for Ov16 antibodies

During June 2021, all study participants were also invited to participate in serological investigations using an Ov16 Rapid Diagnostic Test (RDT, SD Bioline, the Republic of Korea) to determine their exposure to *O. volvulus.* The test was performed following the manufacturer’s instructions. Briefly, the test cassette was labelled with the participant's identification number, and an identified finger was disinfected with an alcohol swab and pricked with the lancet provided. Blood was collected using a capillary tube and placed in the specimen well of the cassette. Four drops of assay diluent were also added to the assay diluent well. All RDT results were read after 20 min.

### Therapeutic and study coverage

Given that in 2009 biannual CDTI started being rolled out in Ghana, we report *therapeutic coverage* data for the first and second treatment rounds in 2009 and 2010 for the study communities where available, calculated as the percent of population treated out of total population in the village (all ages). Where available, we also report therapeutic coverage at implementation unit (IU) level, from Ghana Health Service (GHS) records and the Expanded Special Project for Elimination of NTDs (ESPEN) Ghana portal for Tain and Wenchi, covering a period between 2007 and 2021. The treatment coverage recorded in this study corresponds to the number of people who reported having taken ivermectin in the last treatment round (October) of 2019 and the first treatment round (March) of 2021 divided by the total study population, expressed as a percentage. As the study population is only a fraction of the total population residing in the villages (Table 1), we refer to this coverage as the *study coverage*.

**Table 1:** Description of the socio-demographic information (gender, age, occupation and duration of residence) of study participants

Village	Proportion of village residents recruited for the study, % (*n*/*N*)^e^	Gender, % (*n*/*N*)		Median age (IQR), years	Main occupation (farming), % (*n*/*N*)	Duration of residence in the community (≥ 15 years), % (*n*/*N*)
		Females	Males			
Tain District						
Abekwai 2^a^	48.0% (72/150)	40.3% (29/72)	59.7% (43/72)	19 (11.0‒36.3)	100% (72/72)	47.2% (34/72)
Abekwai 3^a^	26.3% (184/700)	46.2% (85/184)	53.8% (99/184)	24 (15.0‒39.3)	100% (184/184)	59.2% (109/184)
Attakrom^b^	14.1% (113/800)	56.6% (64/113)	43.4% (49/113)	30 (20.0‒45.8)	95.6% (108/113)	42.5% (48/113)
Kokomba^c^	59.2% (71/120)	46.5% (33/71)	53.5% (38/71)	23 (13.0‒38.0)	100% (71/71)	19.7% (14/71)
Wenchi Municipality						
Blibor^d^	23.8% (57/240)	38.6% (22/57)	61.4% (35/57)	23 (15.0‒35.0)	100% (57/57)	42.1% (24/57)
Johnykrom	33.5% (57/170)	36.6% (26/57)	54.4% (31/57)	25 (11.0‒40.0)	94.7% (54/57)	64.9% (37/57)
Subinso 1	7.1% (107/1500)	65.5% (72/107)	33.6% (35/107)	27 (14.3‒44.8)	73.8% (79/107)	71.0% (76/107)
Subinso 2	4.7% (259/5500)	55.0% (142/258)	45.0% (117/258)	30 (18.0‒48.0)	68.7% (178/259)	59.5% (154/259)
Kwanware	34.0% (51/150)	52.9% (27/51)	47.1% (24/51)	26 (9.5‒44.0)	98.0% (50/51)	84.3% (43/51)
Total	10.4% (971/9300)	51.4% (500/971)	48.6% (471/971)	25.2 (9.5‒48.0)	87.8% (853/971)	55.5% (539/971)

^a^Also spelled as Abekwae

^b^Also recorded as Atta-krom

^c^Also spelled as Konkomba

^d^Also recorded as Brilboe

^e^The denominators (*N*) are an approximation provided by the community drug distributors (CDDs) at the time of the study, and do not exactly correspond to the total population sizes reported in the therapeutic coverage records (Additional file 3: Tables S1 and S2)

### Statistical analysis

Continuous variables were summarized using the median and interquartile ranges, and frequencies and percentages were used for categorical variables. The prevalence of epilepsy was calculated by dividing the number of clinically confirmed epilepsy cases by the total number of individuals screened and multiplied by 100. The mean number of treatment rounds in which the participants reported to have taken ivermectin was calculated according to age group. The (crude) community prevalence of microfilarial infection and of Ov16 seropositivity were calculated as the number of positive cases (skin-snip positive or RDT positive) divided by the number examined, expressed as a percentage. 95% *CI* for prevalence values were calculated using the Wilson Score Interval [28]. All statistical analyses were performed using the statistical software Jamovi Desktop 2.3.19.0 (Open-source software, Sydney, Australia) and Graph Pad Prism 8 for macOS 8.2.1 (GraphPad Software, San Diego, CA, USA).

## Results

### Socio-demographic characteristics of participants

This study included a total of 971 participants (from 514 households) consisting of 500 (51.5%) females and 471 (48.5%) males. The median age of participants was 26 years [interquartile range (IQR): 15‒43]. A total of 853 (87.8%) participants reported farming as the main income-generating activity and 539 (55.5%) had lived in the community for ≥ 15 years (Table 1).

### Coverage of ivermectin MDA in the study communities

**Fig. 2:**
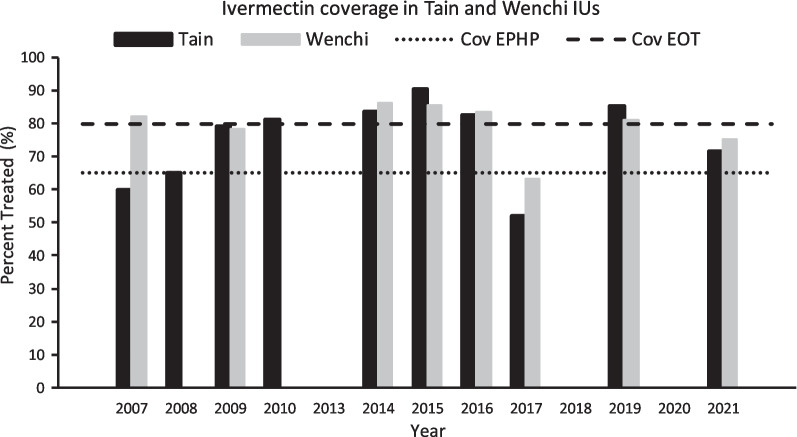
Temporal variation in treatment coverage for Tain and Wenchi implementation units (IUs) from 2007 to 2021. Data for 2007–2010 are from the Ghana Health Service (GHS); data for 2013–2021 are from the ESPEN Ghana portal. Data for 2011–2012 are not available. Treatment was not delivered in 2013 (or not recorded), 2018 and 2020. Dotted line indicates the minimal 65% coverage for elimination of onchocerciasis as a public health problem (Cov EPHP). Dashed line corresponds to enhanced 80% coverage for elimination (interruption) of transmission (Cov EOT)

Additional file 3: Table S1 and S2 present the therapeutic coverage values recorded by the community drug distributors (CDDs) for 2009–2010, and for 2019–2021, respectively. Those recorded in this study (study coverage) for the second treatment round in 2019 and the first treatment round in 2021 are presented in Table 3. There was no CDTI during 2020 due to the interruptions of MDA programmes during the COVID-19 pandemic. Figure 2 presents temporal variation in therapeutic coverage at IU level for Tain and Wenchi from 2007 to 2021. Data for 2011 and 2012 were not available. According to the ESPEN portal, CDTI was not delivered in 2013 (or not recorded), 2018, and as explained above, in 2020.

### Community prevalence of microfilaria infection and of Ov16 seropositivity

In November 2020 (one year after the last 2019 treatment round), the community prevalence of *O.* *volvulus* microfilariae (mf) varied between 4.5% (Abekwai 2) and 11% (Abekwai 3) in the Tain District, and between 2.4% (Subinso 2) and 18.3% (Johnykrom) in the Wenchi Municipality. Across all study communities, the overall mf prevalence was 10.3%. In August 2021 (five months after the treatment round in March), the values remained similar, with an overall mf prevalence of 9.9%. The community prevalence of Ov16 seropositivity ranged from 16.7% (Abekwai 2) to 42.5% (Kokomba) in Tain, and from 10.6% (Blibor) to 13.9% (Johnnykrom) in Wenchi. The overall Ov16 seroprevalence across the communities was 22.2% (29.2% in Tain and 11.9% in Wenchi). In Kwanware, the mf prevalence was 12.3% in 2020 and 11.4% in 2021 (Table 2). Among 34 children aged < 10 years, the prevalence of IgG4 antibodies to the Ov16 antigen was 11.8% (95% *CI*: 4.7–26.6%). The seropositive children were found in Kokomba (2), Johnykrom (1), and Kwanware (1).

**Table 2:** Community microfilarial (mf) prevalence and Ov16 seroprevalence in the nine study communities at the study times of November 2020, and June–August 2021

	Prevalence of mf, % (*n* + ve/*N* examined) [95% *CI*]		Prevalence of Ov16 antibodies, % (*n* + ve/*N* examined) [95% *CI*]
	All participants Nov 2020	All participants Aug 2021	All participants June 2021
Tain District			
Abekwai 2	4.6% (3/66) [1.6–12.5%]	11.9% (5/42) [5.2–25.0%]	16.7% (5/30) [7.3–33.6%]
Abekwai 3	11.0% (8/73) [5.7–20.2%]	4.0% (4/100) [1.6–9.8%%]	31.7% (19/60) [21.3–44.2%]
Attakrom	10.4% (5/48) [4.5–22.2%]	20.7% (6/29) [9.9–38.4%]	22.0% (9/41) [12.0–36.7%]
Kokomba	NA	10.0% (4/40) [4.0–23.1%]	42.5% (17/40) [28.5–57.8%]
Total Tain	8.6% (16/187) [5.3–13.5%]	9.0% (19/211) [5.8–13.6%]	29.2% (50/171) [22.9–36.5%]
Wenchi Municipality			
Blibor	NA	19.5% (8/41) [10.2–34.0%]	10.6% (5/47) [4.6–22.6%]
Johnykrom	18.3% (21/115) [12.3–26.3%]	7.1% (1/14) [1.3–31.5%]	13.9% (5/36) [6.1–28.7%]
Subinso 1	16.2% (16/99) [10.2–24.7%]	8.9% (4/45) [3.5–20.7%]	NA
Subinso 2	2.4% (4/164) [1.0–6.1%]	7.2% (5/69) [3.1–15.9%]	NA
Kwanware	12.3% (9/73) [6.6–21.8%]	11.4% (4/35) [4.5–26.0%]	11.4% (4/35) [4.5–26.0%]
Total Wenchi	11.1% (50/451) [8.5–14.3%]	10.8% (22/204) [7.2–15.8%]	11.9% (14/118) [7.2–18.9%]
Total	10.3% (66/638) [8.2–13.0%]	9.9% (41/415) [7.4–13.1%]	22.2% (64/289) [17.7–27.3%]

*NA* not available (data not collected)

*N* = Number of persons examined in the village; *n* + ve = number with a mf positive skin snip or a positive Ov16 RDT test

In 2020, there was no Community Directed Treatment with Ivermectin because of the COVID-19 pandemic, therefore the data of November 2020 correspond to one year after the last treatment round in October 2019. The data collected in August 2021 correspond to five months after the first treatment round in March 2021.

### Household survey to screen for persons suspected to have epilepsy

The results of the household screening survey to identify persons living with epilepsy (PWE) are presented in Table 3. Fifteen (1.5%, 95% *CI*: 0.0–1.0) participants answered ‘Yes’ when asked whether they were known to have epilepsy. In 5.3% households (27/514), it was reported that someone had previously died from epilepsy. Between 1.3‒2.6% had experienced various symptoms associated with epilepsy including sudden falls with the loss of consciousness, bladder control, foam at the mouth, absence(s) or sudden loss(es) of contact with the surroundings, uncontrollable twitching**,** strange sensations of seeing, hearing and odours. Overall, 63.2% of participants took ivermectin during the last distribution round in March 2021 and the mean number of rounds of ivermectin taken was 7.7 (range: 0–40).

**Table 3:** Results of the household survey to screen for persons suspected to have epilepsy

Variables	Overall (*N* = 971)
Family keeps pigs (*n* = 971)	
No	836 (83.6%)
Yes	135 (13.9%)
A family member died from epilepsy (*n* = 971)	
Don’t know	11 (1.1%)
No	933 (96.1%)
Yes	27 (2.8%)
Known to have epilepsy (*n* = 966)	
Don't know	4 (0.4%)
No	950 (98.0%)
Yes	15 (1.5%)
Sudden fall and loss of consciousness (*n* = 969)	
No	944 (97.4%)
Yes	25 (2.6%)
If yes, did you have loss of bladder control and/ or foam in the mouth (*n* = 25)	
No	7 (28.0%)
Yes	18 (72.0%)
Absence(s) or sudden loss(es) of contact with the surroundings, for a short duration of time (*n* = 969)	
Don't know	1 (0.1%)
No	948 (97.8%)
Yes	20 (2.1%)
Sudden, uncontrollable twitching or shaking of your arms, legs or head, for a period of a few minutes (*n* = 968)	
Don't know	1 (0.1%)
No	945 (97.5%)
Yes	23 (2.4%)
Sudden and brief bodily sensations, see or hear things that are not there, or smell strange odours (*n* = 966)	
Don't Know	3 (0.3%)
No	950 (98.3%)
Yes	13 (1.3%)
Took ivermectin during the last distribution in March 2021 (*n* = 967)	
Don't Know	18 (1.9%)
No	338 (36.8%)
Yes	611 (63.2%)
Mean (range) number of total rounds of ivermectin taken by age groups (*n* = 967)	
5‒30 years	5.21 (0‒20)
31‒60 years	10.3 (0‒40)
> 60 years	14.2 (0‒35)
Overall	7.7 (0‒40)
Epilepsy prevalence according to duration of residence in the community (*n* = 969)	
Resident for < 15 years	1.9% (6/311)
Resident for ≥ 15 years	1.3% (9/658)
Epilepsy prevalence according to District	(*n* = 969)
Tain	1.6% (7/439)
Wenchi	1.5% (8/530)

### Characteristics of the persons with confirmed epilepsy

From the first stage interviews, 20 suspected cases of epilepsy were identified. However, only 14 (70%) were confirmed to have epilepsy. Thus, the prevalence of epilepsy was 1.4% (95% *CI*: 1.0–2.0) (Table 4). The median age of the first seizure in PWE was 12 years (IQR: 6‒14). Most PWEs (92.9%) reported experiencing seizures in the last 5 years and 85.7% in the past 12 months. The most common seizure type was generalized seizures with loss of consciousness (85.7%) with 35.7% reporting a family history of seizures. The most common condition that could be considered as a potential cause of epilepsy was head injury with loss of consciousness (21.4%). The most common anti-seizure medication taken was carbamazepine (35.7%). Among PWE, 64.3% took ivermectin in the last round (March 2021) and 21.4% had never taken ivermectin before. The prevalence of *O. volvulus* microfilariae and Ov16 antibodies in PWE was 8.3% (1/12) and 14.3% (1/7), respectively.

**Table 4:** Clinical characteristics and developmental history of the 14 persons with confirmed epilepsy in the study communities

Characteristics	Overall (*N* = 14)
Females	7 (50%)
Median age, years (IQR)	20 (14‒30)
Symptoms of epilepsy	
Age onset of the first seizure in all PWE, median in years (range)	12 (6‒14 years)
Seizure in the last 5 years, *n* (%)	13 (92.9%)
Aura/sensation before seizures, *n* (%)	10 (71.4%)
Loss of consciousness, *n* (%)	12 (85.7%)
Seizures with passing urine or stool on self and /or foaming at the mouth, *n* (%)	12 (85.7%)
Frequency of seizures, *n* (%)	
Daily (more than 7 per week)	2 (15.4%)
Monthly (less than 4 per month)	4 (30.8%)
Weekly (less than 7 per week)	4 (30.8%)
Yearly (less than 1 per month)	3 (23.1%)
Experienced seizures in the last 12 months?	12 (85.7%)
Reported seizure types, *n* (%)	
Generalized seizures with loss of consciousness	12 (85.7%)
Atonic seizures (drop attacks)	3 (21.4%)
Absences	7 (50.0%)
Focal seizures, consciousness not lost	6 (42.9%)
Focal seizures with decreased consciousness	3 (21.4%)
Family history of seizures, *n* (%)	
Family history of seizures	5 (35.7%)
Father	3 (21.4%)
Mother	2 (14.2%)
Family history of mental illness	3 (21.4%)
Psychomotor development during childhood prior to onset of seizures, *n* (%)	
Normal growth	12 (85.7%)
Learned to do things like other children of his/her age	13 (92.9%)
Cognitive development comparable with peers	10 (71.4%)
Normal mental development	5 (35.7%)
Severe disease preceding onset of seizures, *n* (%)	
Severe measles	2 (14.3%)
Encephalitis/meningitis	1 (7.1%)
Head injury with loss of consciousness	3 (21.4%)
Prolonged post-traumatic coma	1 (7.1%)
Febrile convulsions	2 (14.3%)
Stomach pains and headache	1 (7.1%)
Physical examinations, *n* (%)	
Facial abnormalities	4 (28.6%)
Looks much younger than he/she is (growth retardation)	10 (71.4%)
Burn scars	1 (7.1%)
Itching	4 (28.6%)
Impaired vision	1 (7.1%)
Generalized muscle wasting	4 (28.6%)
Contractures	2 (14.3%)
Neurological examination, *n* (%)	
Alert	10 (71.4%)
Oriented in place/time/person	9 (64.3%)
Paresis	0 (0.0%)
Walks normally	13 (92.9%)
Aggressive episodes	4 (28.6%)
Anti-seizure medication taken, *n* (%)	
Phenobarbital	1 (7.1%)
Carbamazepin	5 (35.7%)
Ethosuximide	1 (7.1%)
Traditional medicines	6 (42.8%)
No anti-seizure medication	6 (42.8%)
Followed up in a health facility for epilepsy treatment	4 (28.6%)
Ivermectin intake, *n* (%)	
Never taken ivermectin	3 (21.4%)
Took ivermectin before onset of seizures	5 (35.7%)
Took ivermectin during the last distribution (March, 2021)	9 (64.3%)
*O. volvulus* infection/exposure, *n/N* (%)	
* O. volvulus* microfilariae	1/12 (8.3%)
Ov16 seropositivity	1/7 (14.3%)

## Discussion

This study aimed at determining the prevalence and clinical characteristics of epilepsy in selected onchocerciasis-endemic communities in the Bono Region of Ghana. Among the study communities, the overall prevalence of *O. volvulus* infection (presence of skin microfilariae) was 10.3% in 2020, and 9.9% in 2021; the overall prevalence of IgG4 antibodies to the Ov16 antigen (Ov16 seroprevalence) was 22.2%, and 11.8% among children aged less than 10 years, indicating that these communities are still endemic for onchocerciasis. In particular, for the village of Kwanware in Wenchi, there is a good temporal record of the evolution of microfilarial prevalence: 48.1% in 1989 (at baseline), 15.6% in 2000 (towards the end of the OCP), 5.6% in 2012 and 29% in 2017 (under CDTI), and 12.3% in 2020 and 11.4% in 2021 (in the present study) (Additional file 3: Figure S1). This trajectory is likely shared by the remaining study villages and suggests that progress towards elimination of onchocerciasis transmission (EOT) has been slow despite the long history of control in the area [29]. Also, the lack of decline in mf prevalence five months after the first 2021 CDTI treatment round is of concern, and suggests that the community residents are not swallowing the ivermectin tablets (despite the good coverage records reported by the CDDs, Additional file 3: Table S2), or are experiencing very fast rates of skin repopulation by mf. Regarding the former, a study on compliance with ivermectin treatment in the study communities will be presented elsewhere. Regarding the latter, a previous study in Ghana (conducted in different communities also located in the middle belt of the country, including the Tain District) identified statistically significantly high mf repopulation rates in some villages which had been recognized as responding sub-optimally to ivermectin [30].

The prevalence of epilepsy in our study communities was 1.4%. This is similar to the median epilepsy prevalence in SSA [31] as well as the average prevalence of epilepsy in Ghana which is estimated at 1% of the population [32]. Expectedly, it is lower than the high epilepsy prevalence reported in onchocerciasis-endemic regions with high pre-control microfilarial prevalence and high ongoing onchocerciasis transmission intensity such as in certain areas in South Sudan (5.1%) [11], Cameroon (3.5% [33] and 7.8% [34], the Democratic Republic of the Congo (4.6% [35] and 3.3% [36]), and Tanzania (2.5%) [37].

This 1.4% prevalence in our study sites is also lower than the 2.9% prevalence for ‘history of convulsions’ reported by community workers in a 2010/2011 study in the demographic surveillance site in Kintampo, an onchocerciasis-endemic area 90‒120 km away from our study districts [20]. However, in Kintampo, after adjusting for attrition and the sensitivity of the screening method, the prevalence of active convulsive epilepsy was only 100.1/1000 (1%). However, given the differences in methodology and epilepsy definitions, we need to compare these epilepsy prevalence findings with great caution. Indeed, we used a two-step screening methodology while in Kintampo a three-step methodology was used which took into account only convulsive epilepsies.

Studies from Uganda [38, 39] and Cameroon [33, 34] have demonstrated that onchocerciasis control/elimination efforts are able to decrease the incidence of epilepsy in an onchocerciasis-endemic area. In the absence of pre-control epilepsy prevalence data, we were unable to demonstrate this in our study, but it is unlikely that the prevalence of OAE was initially high in the study area, or that it would have decreased to the levels observed from higher levels as a result of ivermectin MDA. From the available OCP data, the (standardized) baseline microfilarial prevalence in the Tain and Wenchi study area ranged from 39 to 54%, indicative of mesoendemicity. According to the logistic regression model presented by Pion et al. [40], the corresponding prevalence of OAE would have ranged between 0.6% and 1.2%. According to the EPIONCHO-IBM transmission dynamics model [41] (parameterized for OAE with data published by Chesnais et al. [9]), the prevalence of OAE would have ranged between 1% and 1.5% and the incidence would be approximately 0% after 25 years of ivermectin MDA in mesoendemic settings (Jacob Stapley, pers. comm.).

However, considering the close association between microfilarial prevalence and the prevalence of epilepsy [40], it is important that MDA efforts are improved in the study area in order to ensure that the elimination as a public health problem (EPHP) and EOT goals are achieved. The study recorded a relatively low coverage of ivermectin MDA (63.2% among the general study population and 64.3% among PWE) in 2021, which may be understandable due to the fact that this was the year that CDTI resumed after the COVID-19 pandemic in 2020. Long-term trends may be more informative of the epidemiological situation in the area (Fig. 2), with a number of years in which CDTI has been missed (or not recorded), and therapeutic coverage levels have not consistently reached and maintained the enhanced 80% value recommended for EOT [42] at the IU level. This likely explains why a relatively high prevalence of microfilaridermia persists in the communities (Table 2). A modelling study illustrated the microfilarial rebound that can take place even in long-term programmes when MDA is interrupted [43].

Although, ivermectin coverage has been somewhat variable in the area (Fig. 2), therapeutic coverage has, by and large, surpassed the minimum of 65% recommended for onchocerciasis EPHP [42]. However, the microfilarial prevalence in Tain and Wenchi are at approximately 10% in our study. Although, the coverage of MDA in our study communities is sub-optimal, it is higher than in Owabi in the Ashanti Region of Ghana, where 78.5% of study participants reported that they did not take ivermectin [44]. A study from the Upper Denkyira East Municipality, in the forest area of Ghana, demonstrated that between 2006 and 2013, there was a decline in the mean ivermectin compliance rate from 39% to 26% [45]. Apathy towards MDA programmes is one of the reasons for the low compliance with ivermectin intake. Therefore, there is a need for the National Onchocerciasis Elimination Programme to take immediate and proactive remedial steps to mitigate this challenge, especially as we race towards the EOT targets of 2030 proposed by the World Health Organization (WHO) in its 2021–2030 Roadmap on NTDs [46].

In addition to the interruptions in CDTI experienced both during the COVID-19 pandemic and before it, insufficient funding and human resources, and insufficient guidelines in the past 27 years, have likely hampered the implementation of the Ghana Onchocerciasis Elimination Programme (GOEP). The GOEP has also faced competition for resources from other health programmes, decreasing the buy-in of the government into onchocerciasis control activities [13].

This study had a number of limitations. Recall bias is one of them, as participants/parents/guardians were required to remember their health history and their ivermectin intake. To reduce recall bias, we asked participants to recall their participation in the latest MDA round (March 2021) only 4 months prior to the time when the questionnaires were administered, but we still asked them to try to recall the number of MDA rounds in which they had participated. It is possible that this might not truly reflect the coverage levels of previous years, and future studies should consider this limitation. It is also possible that subtle types of seizures such as absences and some focal seizures with conserved awareness were underreported. Moreover, in our study imaging and laboratory examinations to exclude other causes of epilepsy were not performed. Indeed, with over one-tenth of the population keeping pigs near their homes, other serological and imaging techniques would be required to determine the proportion of individuals with neurocysticercosis as a cause of epilepsy in these communities, and future studies should consider investigating this further. Understanding and mapping the co-endemicity of onchocerciasis and *T. solium* taeniasis/cysticercosis in relation to epilepsy in SSA is a pressing need.

## Conclusions

The epilepsy prevalence in the Bono region was 1.4% which is similar to the median epilepsy prevalence in SSA. Also, *O. volvulus* microfilariae were found in only one of the 12 PWE tested during skin snip examination. Both findings mean that currently in the Bono region there is no appreciable OAE. However, despite 27 years of ivermectin administration, the overall mf prevalence in the community is still around 10%. Therefore, there is a need for the GOEP to strengthen and sustain its efforts to ensure that the gains of MDA are consolidated and improved to achieve the elimination of onchocerciasis and its associated morbidities, and importantly, to reach the 2030 goals proposed by the WHO [46].

### Supplementary Information

**Additional file 1**: First-stage household questionnaire.

**Additional file 2**: Second-stage neurological questionnaire.

**Additional file 3**: Coverage data and temporal microfilarial trends.

## Data Availability

All the data are contained in the Tables, Figures, and Additional files.

## Abbreviations

APOC: African Programme for Onchocerciasis Control
CDTI: Community-directed treatment with ivermectin
CDD: Community drug distributor
*CI*: Confidence interval
Cov EPHP: Coverage for Elimination as Public Health Problem
Cov EOT: Coverage for Elimination of Transmission
COVID-19: Coronavirus disease 2019
EOT: Elimination (interruption) of transmission
EPHP: Elimination as a Public Health Problem
ESPEN: Expanded Special Project for the Elimination of Neglected Tropical Diseases
GHS: Ghana Health Service
GOEP: Ghana Onchocerciasis Elimination Programme
ILAE: International League Against Epilepsy
IU: Implementation unit
LMIC: Low- and middle-income countries
LF: Lymphatic filariasis
MDA: Mass drug administration
Mf: Microfilariae
NTD: Neglected Tropical Diseases
OAE: Onchocerciasis-associated epilepsy
OCP: Onchocerciasis Control Programme in West Africa
*OR*: Odds ratio
PWE: Persons living with epilepsy
RDT: Rapid diagnostic test
REMO: Rapid Epidemiological Mapping of Onchocerciasis
SIZ: Special Intervention Zone
SSA: Sub-Saharan Africa
WHO: World Health Organization

## References

[1] Frallonardo L, Di Gennaro F, Panico GG, Novara R, Pallara E, Cotugno S (2022). Onchocerciasis: current knowledge and future goals. Front Trop Dis.

[2] Duke BOL (1990). Human onchocerciasis—an overview of the disease. Acta Leiden.

[3] Otache AE, Ezenwosu IL, Ossai EN, Aniwada EC, Abah SO, Uzochukwu BC (2022). Disability and its determinants among individuals with onchocerciasis in Southeast Nigeria: a cross-sectional study. Pan Afr Med J.

[4] Basáñez MG, Pion SDS, Churcher TS, Breitling LP, Little MP, Boussinesq M (2006). River blindness: a success story under threat?. PLoS Med.

[5] Coffeng LE, Stolk WA, Zouré HGM, Veerman JL, Agblewonu KB, Murdoch ME (2013). African programme for onchocerciasis control 1995–2015: model-estimated health impact and cost. PLoS Negl Trop Dis.

[6] Colebunders R, Siewe Fodjo JN, Hopkins A, Hotterbeekx A, Lakwo TL, Kalinga A (2019). From river blindness to river epilepsy: implications for onchocerciasis elimination programmes. PLoS Negl Trop Dis.

[7] Colebunders R, Njamnshi AK, Menon S, Newton CR, Hotterbeekx A, Preux PM (2021). *Onchocerca volvulus* and epilepsy: a comprehensive review using the Bradford Hill criteria for causation. PLoS Negl Trop Dis.

[8] Galán-Puchades MT (2019). Onchocerciasis-associated epilepsy. Lancet Infect Dis.

[9] Chesnais CB, Nana-Djeunga HC, Njamnshi AK, Lenou-Nanga CG, Boullé C, Bissek ACZK (2018). The temporal relationship between onchocerciasis and epilepsy: a population-based cohort study. Lancet Infect Dis.

[10] Mukendi D, Tepage F, Akonda I, Siewe JNF, Rotsaert A, Ndibmun CN (2019). High prevalence of epilepsy in an onchocerciasis endemic health zone in the Democratic Republic of the Congo, despite 14 years of community-directed treatment with ivermectin: a mixed-method assessment. Int J Infect Dis.

[11] Raimon S, Dusabimana A, Abd-Elfarag G, Okaro S, Carter JY, Newton CR (2021). High prevalence of epilepsy in an onchocerciasis-endemic area in Mvolo county, South Sudan: a door-to-door survey. Pathogens.

[12] Alley ES, Plaisier AP, Boatin BA, Dadzie KY, Remme J, Zerbo G (1994). The impact of five years of annual ivermectin treatment on skin microfilarial loads in the onchocerciasis focus of Asubende, Ghana. Trans R Soc Trop Med Hyg.

[13] Biritwum NK, de Souza DK, Asiedu O, Marfo B, Amazigo UV, Gyapong JO (2021). Onchocerciasis control in Ghana (1974–2016). Parasit Vectors.

[14] Stolk WA, Borsboom GJJM, Habbema JDF. Trends in infection prevalence in the Special Intervention Zones of the former OCP and future expectations: Final Report. 2011. Erasmus Medical Center, Rotterdam, The Netherlands.

[15] Biritwum NK, de Souza DK, Marfo B, Odoom S, Alomatu B, Asiedu O (2017). Fifteen years of programme implementation for the elimination of lymphatic filariasis in Ghana: impact of MDA on immunoparasitological indicators. PLoS Negl Trop Dis.

[16] Noma M, Nwoke BEB, Nutall I, Tambala PA, Enyong P, Namsenmo A (2002). Rapid epidemiological mapping of onchocerciasis (REMO): Its application by the African Programme for Onchocerciasis Control (APOC). Ann Trop Med Parasitol.

[17] Le Berre R, Garms R, Davies JB, Walsh JF, Philippon B (1979). Displacements of *Simulium damnosum* and strategy of control against onchocerciasis. Philos Trans R Soc London Ser B Biol Sci.

[18] Baker RHA, Guillet P, Sékétéli A, Poudiougo P, Boakye D, Wilson MD (1990). Progress in controlling the reinvasion of windborne vectors into the Western area of the Onchocerciasis Control Programme in West Africa [and Discussion]. Philos Trans R Soc B Biol Sci.

[19] Turner HC, Osei-Atweneboana MY, Walker M, Tettevi EJ, Churcher TS, Asiedu O (2013). The cost of annual versus biannual community-directed treatment of onchocerciasis with ivermectin: Ghana as a case study. PLoS Negl Trop Dis.

[20] Ae-Ngibise KA, Akpalu B, Ngugi A, Akpalu A, Agbokey F, Adjei P (2015). Prevalence and risk factors for active convulsive epilepsy in Kintampo, Ghana. Pan Afr Med J.

[21] Ghana Statistical Service (2014). 2010 Population and Housing Census. District Analytical Report. Tain District. https://www2.statsghana.gov.gh/docfiles/2010_District_Report/Brong%20Ahafo/TAIN.pdf. Accessed 24 Apr 2023.

[22] Ghana Statistical Service (2014). 2010 Population and Housing Census. District Analytical Report. Wenchi Municipality. https://www2.statsghana.gov.gh/docfiles/2010_District_Report/Brong%20Ahafo/WENCHI.pdf. Accessed 24 Apr 2023.

[23] Bharucha N, Odermatt P, Preux PM (2013). Methodological difficulties in the conduct of neuroepidemiological studies in low- and middle-income countries. Neuroepidemiology.

[24] Diagana M, Preux PM, Tuillas M, Hamady AO, Druet-Cabanac M (2006). Dépistage de l’épilepsie en zones tropicales: validation d’un questionnaire en Mauritanie. Bull Soc Pathol Exot.

[25] Fisher RS, Acevedo C, Arzimanoglou A, Bogacz A, Cross JH, Elger CE (2014). ILAE Official Report: a practical clinical definition of epilepsy. Epilepsia.

[26] KoboCollect. KoboToolBox. Apps on Google Play. 2020. https://play.google.com/store/apps/details?id=org.koboc.collect.android&hl=en&gl=US&pli=1. Accessed 24 Apr 2023.

[27] Otabil KB, Gyasi SF, Awuah E, Obeng-Ofori D, Atta-Nyarko RJ, Andoh D (2019). Prevalence of onchocerciasis and associated clinical manifestations in selected hypoendemic communities in Ghana following long-term administration of ivermectin. BMC Infect Dis.

[28] Brown LD, Cai TT, DasGupta A (2001). Interval estimation for a binomial proportion. Stat Sci.

[29] World Health Organization. Guidelines for stopping mass drug administration and verifying elimination of human onchocerciasis: Criteria and procedures. Geneva: World Health Organization. 2016. http://apps.who.int/iris/bitstream/10665/204180/1/9789241510011_eng.pdf?ua=1. Accessed 24 Apr 2023. 26913317

[30] Frempong KK, Walker M, Cheke RA, Tetevi EJ, Gyan ET, Owusu EO (2016). Does increasing treatment frequency address suboptimal responses to ivermectin for the control and elimination of river blindness?. Clin Infect Dis.

[31] Ba-Diop A, Marin B, Druet-Cabanac M, Ngoungou EB, Newton CR, Preux PM (2014). Epidemiology, causes, and treatment of epilepsy in sub-Saharan Africa. Lancet Neurol.

[32] Ministry of Health Ghana, World Health Organization. “Fight Against Epilepsy” Initiative in Ghana. WHO Programme on reducing the epilepsy treatment gap 2012‒2016. 2018. https://www.afro.who.int/sites/default/files/2018-11/WHO-Epilepsy-Ghana_web.pdf. Accessed 24 April 2023.

[33] Boulle C, Njamnshi AK, Dema F, Mengnjo MK, SieweFodjo JN, Bissek ACZK (2019). Impact of 19 years of mass drug administration with ivermectin on epilepsy burden in a hyperendemic onchocerciasis area in Cameroon. Parasit Vectors..

[34] Siewe Fodjo JN, Tatah G, Tabah EN, Ngarka L, Nfor LN, Chokote SE (2018). Epidemiology of onchocerciasis-associated epilepsy in the Mbam and Sanaga river valleys of Cameroon: impact of more than 13 years of ivermectin. Infect Dis Poverty.

[35] Lenaerts E, Mandro M, Mukendi D, Suykerbuyk P, Dolo H, Wonya’ Rossi D (2018). High prevalence of epilepsy in onchocerciasis endemic health areas in Democratic Republic of the Congo. Infect Dis Poverty.

[36] Levick B, Laudisoit A, Tepage F, Ensoy-Musoro C, Mandro M, Bonareri Osoro C (2017). High prevalence of epilepsy in onchocerciasis endemic regions in the Democratic Republic of the Congo. PLoS Negl Trop Dis.

[37] Mmbando BP, Suykerbuyk P, Mnacho M, Kakorozya A, Matuja W, Hendy A (2018). High prevalence of epilepsy in two rural onchocerciasis endemic villages in the Mahenge area, Tanzania, after 20 years of community-directed treatment with ivermectin. Infect Dis Poverty.

[38] Gumisiriza N, Kaiser C, Asaba G, Onen H, Mubiru F, Kisembo D (2020). Changes in epilepsy burden after onchocerciasis elimination in a hyperendemic focus of western Uganda: a comparison of two population-based, cross-sectional studies. Lancet Infect Dis.

[39] Gumisiriza N, Mubiru F, Siewe Fodjo JN, Mbonye Kayitale M, Hotterbeekx A, Idro R (2020). Prevalence and incidence of nodding syndrome and other forms of epilepsy in onchocerciasis-endemic areas in northern Uganda after the implementation of onchocerciasis control measures. Infect Dis Poverty.

[40] Pion SDS, Kaiser C, Boutros-Toni F, Cournil A, Taylor MM, Meredith SE (2009). Epilepsy in onchocerciasis endemic areas: systematic review and meta-analysis of population-based surveys. PLoS Negl Trop Dis.

[41] Hamley JID, Milton P, Walker M, Basáñez MG (2019). Modelling exposure heterogeneity and density dependence in onchocerciasis using a novel individual-based transmission model, EPIONCHO-IBM: implications for elimination and data needs. PLoS Negl Trop Dis.

[42] World Health Organization & African Programme for Onchocerciasis Control. Report of the CSA (Committee of Sponsoring Agencies) Advisory Group on onchocerciasis elimination. 2011. Joint Action Forum 17th Session (JAF 17.4 ii), Kuwait City, Kuwait, 12‒14 December 2011. https://apps.who.int/iris/handle/10665/347055. Accessed 24 Apr 2023.

[43] Hamley JID, Blok DJ, Walker M, Milton P, Hopkins AD, Hamill LC (2021). What does the COVID-19 pandemic mean for the next decade of onchocerciasis control and elimination?. Trans R Soc Trop Med Hyg.

[44] Osei Agyemang AN, Badu K, Baffour-Awuah S, Owusu-Dabo E, Biritwum NK, Garms R (2018). Evaluation of onchocerciasis control in the Upper Denkyira East municipal in the forest area of Ghana: responses of participants and distributors to the CDTI programme. Acta Trop.

[45] Osei FA, Newton S, Nyanor I, Osei-Yeboah E, Amuzu EX, Mensah NK (2022). Mass drug administration targeting *Onchocerca volvulus* in Owabi catchment area in Ashanti Region, Ghana: determinants of drug coverage and drug uptake. Parasite Epidemiol Control.

[46] World Health Organization. Ending the neglect to attain the Sustainable Development Goals: a road map for Neglected Tropical Diseases 2021–2030. 2021. Geneva: World Health Organization. https://www.who.int/publications/i/item/9789240010352. Accessed 24 Apr 2023.

