# Supplementary material for: Prevalence of epilepsy in the onchocerciasis endemic middle belt of Ghana after 27 years of mass drug administration with ivermectin
Source: Infect Dis Poverty. 2023 Aug 17;12:75. doi: 10.1186/s40249-023-01117-9 (PMC10433588; doi:10.1186/s40249-023-01117-9)
Supplement: Supplementary file 1 — Additional file 1: First-stage household questionnaire. [file 40249_2023_1117_MOESM1_ESM.docx]

**Additional file 1: Household-level questionnaire**

**Prevalence of epilepsy in the onchocerciasis endemic middle belt of Ghana after 27 years of Mass Drug Administration with ivermectin**

Kenneth Bentum Otabil^1,2,3*^, Blessing Ankrah^1^, Emmanuel John Bart-Plange^1,4^, Emmanuel Sam Donkoh^4^, Fiona Amoabil Avarikame^4^, Fredrick Obeng Ofori-Appiah^4^, Theophilus Nti Babae^1^, Prince-Charles Kudzordzi^1^, Vera Achiaa Darko^1,5^, Joseph Ameyaw^6^, Joseph Gyekye Bamfo^7^, Abdul Sakibu Raji^2^, Daniel Antwi-Berko^4^, Joseph Nelson Siewe Fodjo^3^, María-Gloria Basáñez^8^, Henk D F H Schallig^9^, Robert Colebunders^3^

^1^NeTroDis Research Group, Centre for Research in Applied Biology, School of Sciences, University of Energy and Natural Resources, Sunyani, Bono Region, Ghana

^2^Department of Biological Science, School of Sciences, University of Energy and Natural Resources, Sunyani, Bono Region, Ghana

^3^Global Health Institute, University of Antwerp, Belgium

^4^Department of Medical Laboratory Science, School of Sciences, University of Energy and Natural Resources, Sunyani, Bono Region, Ghana

^5^STU Clinic, Sunyani Technical University, Sunyani, Bono Region, Ghana

^6^Happy Family Hospital, Nkoranza, Bono East Region

^7^Tain District Hospital, Nsawkaw, Bono East Region, Ghana

^8^MRC Centre for Global Infectious Disease Analysis (MRC GIDA), and London Centre for Neglected Tropical Disease Research, Department of Infectious Disease Epidemiology, School of Public Health, Imperial College London, London, UK

^9^Amsterdam University Medical Centres, Academic Medical Centre at the University of Amsterdam, Department of Medical Microbiology, Experimental Parasitology Unit, Amsterdam, The Netherlands

* **Corresponding author:** Kenneth Bentum Otabil, E**-**mail: [**kenneth.otabil@uenr.edu.gh**](mailto:kenneth.otabil@uenr.edu.gh)

***Household level Questionnaire***

*NOTE: This questionnaire will be administered in a digitalized form using the Kobo-collect software on a tablet computer that will be used as a data collection tool during this survey. Within each household, all participants will be asked all the questions in sections 1.2 to 1.3 during one single interview sequence.*

DATE: _____ / _____ / ________

FULL NAME OF INTERVIEWER: ________________________________________

DISTRICT _________________________________________

VILLAGE: _________________________________________

### HOUSEHOLD CHARACTERISTICS

UNIQUE HOUSEHOLD CODE:
XX/XX /XX/XXXX (First 2 letters of: State/County/Payam/Village, followed by number (e.g.0001))

GPS coordinates of household:
LATITUDE __ __. __ __ __ __LONGITUDE __ __. __ _____ __ ALTITUDE _ __ __ __

Full name of household head: _____________________________________
Mobile phone number of household head: ___________________________________
Ethnic group of household head: __________________________________________

Duration the family lives in the village _______________________

Total number of people in the household: ___________________________________

Livelihood generating activities of the family:

Farmer  Livestock breeder  Fisherman  Civil service
 Business/trader  Craftsman  Other, specify:_________________

Does the family keep pigs YES  NO

Has there been a family member that died from any form of epilepsy? YES NO DON’T KNOW

IF YES: When (year) ________ At what age: ________ years
IF MORE THAN ONE: When (year) ________ At what age: _____ years

### INDIVUDUAL INTERVIEW WITH EACH HOUSEHOLD MEMBER

Personal ID: __________________________________________
Full Name: __________________________________________
Age: __________years
Date of birth: ___/___/____
Sex: Male Female

Is the person present during the interview visit? YES  NO
Who is answering to the questions?  Self  Mother  Household head  Other, specify ________________

### SCREENING FOR EPILEPSY

*If at least one of the 5 questions is answered with YES, the electronic questionnaire will automatically report the person for invitation to participate in the neurological survey for case verification.*

**QUESTION A.**Is the participant / are you suffering from nodding syndrome?

YES NO DON’T KNOW

**QUESTION B.**Is the participant / are you suffering from epilepsy?

YES NO DON’T KNOW

**QUESTION C.**Is the person known to suffer from both nodding syndrome and epilepsy?

YES NO DON’T KNOW

**QUESTION 1.**Have you ever suddenly fallen to the ground and experienced:

1. Loss of consciousness YES NO DON’T KNOW
2. Loss of bladder control? YES NO DON’T KNOW

c) Foam at the mouth? YES NO DON’T KNOW

**QUESTION 2.**Have you ever experienced absence(s) or sudden loss(es) of contact with the surroundings, for a short duration of time? YES NO DON’T KNOW

**QUESTION 3.**Have you ever experienced sudden, uncontrollable twitching or shaking of your arms, legs or head, for a period of a few minutes? YES NO DON’T KNOW

**QUESTION 4.**
Do you sometimes experience sudden and brief bodily sensations, see or hear things that are not there, or smell strange odors? YES NO DON’T KNOW

**QUESTION 5.**Have you ever been told that you are suffering from epilepsy or have you already experienced **at least one episode of seizures**? YES NO DON’T KNOW

### IVERMECTIN USE

How many years did you took ivermectin? Times: __________________

Did you take ivermectin during the last distribution?YES NO DON’T KNOW

If YES, when? Year (s)_________, __________, _________
