# Supplementary material for: Prevalence of epilepsy in the onchocerciasis endemic middle belt of Ghana after 27 years of mass drug administration with ivermectin
Source: Infect Dis Poverty. 2023 Aug 17;12:75. doi: 10.1186/s40249-023-01117-9 (PMC10433588; doi:10.1186/s40249-023-01117-9)
Supplement: Supplementary file 2 — Additional file 2: Second-stage neurological questionnaire. [file 40249_2023_1117_MOESM2_ESM.docx]

**Additional file 2: Neurological Questionnaire**

**Prevalence of epilepsy in the onchocerciasis endemic middle belt of Ghana after 27 years of Mass Drug Administration with ivermectin**

Kenneth Bentum Otabil^1,2,3*^, Blessing Ankrah^1^, Emmanuel John Bart-Plange^1,4^, Emmanuel Sam Donkoh^4^, Fiona Amoabil Avarikame^4^, Fredrick Obeng Ofori-Appiah^4^, Theophilus Nti Babae^1^, Prince-Charles Kudzordzi^1^, Vera Achiaa Darko^1,5^, Joseph Ameyaw^6^, Joseph Gyekye Bamfo^7^, Abdul Sakibu Raji^2^, Daniel Antwi-Berko^4^, Joseph Nelson Siewe Fodjo^3^, María-Gloria Basáñez^8^, Henk D F H Schallig^9^, Robert Colebunders^3^

^1^NeTroDis Research Group, Centre for Research in Applied Biology, School of Sciences, University of Energy and Natural Resources, Sunyani, Bono Region, Ghana

^2^Department of Biological Science, School of Sciences, University of Energy and Natural Resources, Sunyani, Bono Region, Ghana

^3^Global Health Institute, University of Antwerp, Belgium

^4^Department of Medical Laboratory Science, School of Sciences, University of Energy and Natural Resources, Sunyani, Bono Region, Ghana

^5^STU Clinic, Sunyani Technical University, Sunyani, Bono Region, Ghana

^6^Happy Family Hospital, Nkoranza, Bono East Region

^7^Tain District Hospital, Nsawkaw, Bono East Region, Ghana

^8^MRC Centre for Global Infectious Disease Analysis (MRC GIDA), and London Centre for Neglected Tropical Disease Research, Department of Infectious Disease Epidemiology, School of Public Health, Imperial College London, London, UK

^9^Amsterdam University Medical Centres, Academic Medical Centre at the University of Amsterdam, Department of Medical Microbiology, Experimental Parasitology Unit, Amsterdam, The Netherlands

* **Corresponding author:** Kenneth Bentum Otabil, E**-**mail: [**kenneth.otabil@uenr.edu.gh**](mailto:kenneth.otabil@uenr.edu.gh)

### Neurology questionnaire

DATE: _____ / _____ / ________

FULL NAME OF THE SPECIALISED HEALTH CARE WORKER: _______________

### PARTICIPANT IDENTIFICATION

Participant ID _____________________________________

Last name *(in capital letters)*: _____________________________________

First name: __________________________________________

### PARTICIPANT IDENTIFICATION

Participant ID _____________________________________

Last name *(in capital letters)*: _____________________________________

First name: __________________________________________

Address *(any information that allows locating the individual)*: ____________________

Town / Village: __________________________________________

State: _______________________________________________
Phone number: __________________________________________
Sex: Male Female

Age: _______________years
Date of birth: ___/___/____

Place of birth: _____________________________________

Country of birth: __________________________________________
Ethnic group: __________________________________________

Marital status: Married Living with parents In partnership
 Living alone other, specify _________________

Is the participant answering himself / herself?  YES  NO

If NO, who is answering and what is the relationship between the participant and respondent?
Full name: __________________________________________
Relation to participant: Mother Father Sibling other, specify _____________________________

### DEMOGRAPHIC DATA

Since how long is the participant lived in the village? ___________YEARS

IF less than 1 year: how many MONTHS? ______________

### HISTORY OF EPILEPSY

Has the participant had a seizure in the last 5 years?

YES  NO  DON’T KNOW

What is the number of seizures since onset?

One attack  Two or more seizures

If only two seizures, were they more than 24h apart?  YES  NO  DON’T KNOW

Aura / sensation (hearing, seeing, tasting, smelling, feeling) before seizures:  YES  NO  DON’T KNOW

Episodes of loss of consciousness  YES  NO  DON’T KNOW

Seizures with passing urine or stool on self and /or foaming at the mouth

YES  NO  DON’T KNOW

Has he the participant had a seizure within the last 12 months?

YES  NO  DON’T KNOW

IF YES, in which month has the last seizure been experienced?
 MONTH: _ _  DON’T KNOW

What is the current frequency of the seizures?

Yearly (if less than 1 per month)

Monthly (if less than 4 per month)

Weekly (if less than 7 per week)

Daily (if more than 7 per week)

Specify number: _____________per ______________

How many seizures did you have LAST WEEK?  number____  None  DON’T KNOW

Was the onset of seizures within the first year of life?
  YES  NO  DON’T KNOW

IF the onset of the seizures was within the first year of life:
  During the first 10 days of life
  More than 10 days to 6 month
  More than 6 month to 1 year

If the onset of seizures was after the age of one, at what age?
 __________ years (try to estimate if not known exactly)

What was the type of the initial seizures? (different types of seizures possible)
  Generalized seizures with loss of consciousness
  Atonic seizures (drop attacks)
  Absences

Nodding seizures
  Focal seizures, consciousness not lost
  Focal seizures with decreased consciousness
  Secondarily generalized bilateral seizures
  Others, specify:___________________________

What type are currently the seizures? (different types of seizures possible)
  Generalized seizures with loss of consciousness
  Atonic seizures (drop attacks)
  Absences

Nodding seizures
  Focal seizures, consciousness not lost
  Focal seizures with decreased consciousness
  Secondarily generalized bilateral seizures
  Others, specify:___________________________

### MEDICAL HISTORY

Family history of seizures  YES  NO  DON’T KNOW

IF YES, specify who these are (tick all that apply)

Siblings (brother/sister); No. of affected siblings ______
 Father Mother Grandparent(s)

Family history of mental illness  YES  NO  DON’T KNOW

***Questions for the mother of the participant***

Did the pregnancy of the mother of the participant proceed normally?

YES  NO  DON’T KNOW
If NO, specify: ___________________________________

Mode of delivery for the affected child:

Spontaneous Vaginal Delivery
  Assisted Vaginal Delivery

Caesarean section

Was the interviewed participant born at term (pregnancy had completed 9 months)?

YES  NO  DON’T KNOW

Did the interviewed participant cry immediately after birth?

YES  NO  DON’T KNOW

What was the birth weight? ______________________GRAMS  DON’T KNOW

***Psychomotor Development during Childhood:***

**Prior to onset of seizures**

Was the child growing normally prior to the onset of the seizures?
  Yes  No  DON’T KNOW NAP

IF NO, at what age did the abnormal growing appear? ______ years

Did the child learn to do things like other children of his/her age prior to the onset of the seizures?  Yes  No  DON’T KNOW NAP

IF NO, at what age did the learning difficulty start? ______ year

Compared with other children of his/her age, did the child appear in any way mentally backward, dull or slow before the onset of the seizures?  Yes  No  DON’T KNOW NAP

IF YES, at what age did it start? ______ Years

**Since the onset of seizures**,

Compared with other children of his/her age, did the child learned to do things like other children?

NAP Normal  Delayed  Abnormal
  Others, specify:___________________________

Compared with other children of his/her age, did the child appear in any way mentally backward, dull or slow?

NAP Normal  Delayed  Abnormal
  Others, specify:__________________________

***Occurrence of severe disease in the past:***

Has the interviewed participant suffered from severe measles before the onset of epileptic seizures?
 YES  NO  DON’T KNOW

If Yes how long before the onset of epilepsy ………………. Years  less than 1 year

Has the interviewed participant suffered from a severe form of malaria before the onset of epileptic seizures?
 YES  NO  DON’T KNOW

If Yes how long before the onset of epilepsy ………………. Years  less than 1 year

Has the interviewed participant suffered from encephalitis/meningitis before the onset of epileptic seizures?
 YES  NO  DON’T KNOW

If Yes how long before the onset of epilepsy ………………. Years  less than 1 year

Has the participant had a head injury with loss of consciousness before the onset of epileptic seizures?
 YES  NO  DON’T KNOW

If Yes how long before the onset of epilepsy ………………. Years  less than 1 year

Has the participant had a prolonged post-traumatic coma before the onset of epileptic seizures?
 YES  NO  DON’T KNOW

If Yes how long before the onset of epilepsy ………………. Years  less than 1 year

Has the patient presented febrile convulsions in the past?  YES  NO  DON’T KNOW

If Yes how long before the onset of epilepsy ……………….Years  less than 1 year

Was the onset of epilepsy following another illness?
 YES  NO  DON’T KNOW
If YES, specify the illness: _______________________________

If Yes how long before the onset of epilepsy ……………….Years  less than 1 year

Has the participant has a history of excessive alcohol consumption?
 YES  NO  DON’T KNOW  Not applicable*

Has the participant has a history of drugs abuse?
YES  NO  DON’T KNOW  Not applicable*

### GENERAL EXAMINATION Done Not done

BODY WEIGHT (kg): _________ . ____kg

HEIGHT (cm): _______________cm

How is the general condition of the interviewed participant?

GOOD CORRECT POOR

Thoracic abnormalities  YES  NO

If yes specify ________________________________________

Facial abnormalities  YES  NO

If yes specify ____________________________________________

Does the person look much younger than he/she is?  YES  NO

If yes and if the person > 16 years are there external signs of sexual development conform to age:

YES  NO  EXAMINATION DECLINED

If NO, specify: girls:  Breast not developed
girls and boys:  No pubic hear

Ophthalmology  NORMAL  ABNORMAL VISION
 BLIND ONE EYE  BOTH EYES AFFECTED

Burn scars  YES  NO

Itching  YES  NO

Papular nodular eruption  YES  NO

Depigmented lesions (leopard skin)  YES  NO

Dry, thick wrinkled skin (lizard skin)  YES  NO

Suspected onchocerciasis nodules  YES  NO

If yes, number of nodules __________________

### NEUROLOGICAL EXAMINATION

Is the participant alert?  YES  NO

Fully oriented in place/time/person  YES  NO

Is the participant’s cognitive development comparable with peers?
  YES  NO

Normal vision  YES  NO

Normal hearing  YES  NO

Normal eye movements?  YES  NO

Generalized muscle wasting  YES  NO

Paresis  YES  NO

if YES specify_________________________________________________________

Contractures  YES  NO

Is the participant walking normally?  YES  NO

IF NO, specify  Ataxic (wide base) gait

Waddling gait (like a duck)

Spastic gait –with tip toe walking

Hemiplegic – with one sided weakness

Other, specify______________________

***Psychiatric symptoms***

Does the participant show aggressive episodes?  YES  NO

Does the participant suffer from another neuro-psychiatric / psychological problem?
 YES  NO  DON’T KNOW

IF YES, specify_______________________________

***Physical / Functional Indices:*** *Please mark the most accurate description of the current functional state of the person with epilepsy, as observed during the evaluation*

| 1 | No significant disability despite symptoms; able to carry out all usual duties and activities |
| --- | --- |
| 2 | Slight disability; unable to carry out all previous activities, but able to look after own affairs without assistance |
| 3 | Moderate disability; requiring some help, but able to walk without assistance |
| 4 | Moderately severe disability; unable to walk without assistance and unable to attend to own bodily needs without assistance |
| 5 | Severe disability; bedridden, incontinent and requiring constant nursing care and attention |

### CASE CLASSIFICATION

Epilepsy
  Other diagnosis

If other diagnosis:
  Recurrent febrile convulsions

Dizziness / syncope

Paroxysmal vertigo

Severe anaemia

Mental retardation without epilepsy
  Psychiatric illness without epilepsy

Classic migraine

Only one seizure

Other, specify_________

### TREATMENT

What is or was the type of seizure medication taken by the participant?

No treatment  DON’T KNOW
  Traditional  anti-epileptic drug
  Mixed

*If anti-epileptic drug treatment:* Which substance is taken by the participant (additionally you may check patient’s treatment record)?

Phenytoin  YES  NO if yes dose ____________
Sodium valproate  YES  NO if yes dose ____________
Phenobarbital  YES  NO if yes dose ____________
Carbamazepin  YES  NO if yes dose ____________
Diazepam  YES  NO if yes dose ____________
Ethosuximide  YES  NO if yes dose ____________
Other anti-epileptic treatment  YES  NO if yes dose ____________
If YES, specify: _______________________________________

*Compliance*: Is the participant taking the anti-epileptic drug treatment regularly?
  YES  NO
If NO, why?  Side effects
  (Temporary) non-availability of medication
  Lack of financial means to access medication
  DON’T KNOW
  Other, specify ____________________________

Are you currently in followed up in a health centre for treatment of epilepsy:

YES  NO

If yes, specify where______________________________________________

1.2.10 IVERMECTIN USE

Has the participant ever received ivermectin?

YES  NO  DON’T KNOW

NOT APPLICABLE (according to exclusion criteria, as follows):

If yes, how many years: _____________years

Did the person took ivermectin before the development of the seizures YES  NO  DON’T KNOW

Did the person take ivermectin during the last distribution_____________?

**Action Taken by reporting officer**: referred for treatment  YES NO

If yes, where? _________________________________________________________
