# Supplementary material for: Prevalence of epilepsy in the onchocerciasis endemic middle belt of Ghana after 27 years of mass drug administration with ivermectin
Source: Infect Dis Poverty. 2023 Aug 17;12:75. doi: 10.1186/s40249-023-01117-9 (PMC10433588; doi:10.1186/s40249-023-01117-9)
Supplement: Supplementary file 3 — Additional file 3: Coverage data and temporal microfilarial trends. [file 40249_2023_1117_MOESM3_ESM.docx]

**Additional file 3: Coverage data and temporal microfilarial trends**

**Prevalence of epilepsy in the onchocerciasis endemic middle belt of Ghana after 27 years of Mass Drug Administration with ivermectin**

Kenneth Bentum Otabil^1,2,3*^, Blessing Ankrah^1^, Emmanuel John Bart-Plange^1,4^, Emmanuel Sam Donkoh^4^, Fiona Amoabil Avarikame^4^, Fredrick Obeng Ofori-Appiah^4^, Theophilus Nti Babae^1^, Prince-Charles Kudzordzi^1^, Vera Achiaa Darko^1,5^, Joseph Ameyaw^6^, Joseph Gyekye Bamfo^7^, Abdul Sakibu Raji^2^, Daniel Antwi-Berko^4^, Joseph Nelson Siewe Fodjo^3^, María-Gloria Basáñez^8^, Henk D F H Schallig^9^, Robert Colebunders^3^

^1^NeTroDis Research Group, Centre for Research in Applied Biology, School of Sciences, University of Energy and Natural Resources, Sunyani, Bono Region, Ghana

^2^Department of Biological Science, School of Sciences, University of Energy and Natural Resources, Sunyani, Bono Region, Ghana

^3^Global Health Institute, University of Antwerp, Belgium

^4^Department of Medical Laboratory Science, School of Sciences, University of Energy and Natural Resources, Sunyani, Bono Region, Ghana

^5^STU Clinic, Sunyani Technical University, Sunyani, Bono Region, Ghana

^6^Happy Family Hospital, Nkoranza, Bono East Region

^7^Tain District Hospital, Nsawkaw, Bono East Region, Ghana

^8^MRC Centre for Global Infectious Disease Analysis (MRC GIDA), and London Centre for Neglected Tropical Disease Research, Department of Infectious Disease Epidemiology, School of Public Health, Imperial College London, London, UK

^9^Amsterdam University Medical Centres, Academic Medical Centre at the University of Amsterdam, Department of Medical Microbiology, Experimental Parasitology Unit, Amsterdam, The Netherlands

* **Corresponding author:** Kenneth Bentum Otabil, E**-**mail: [**kenneth.otabil@uenr.edu.gh**](mailto:kenneth.otabil@uenr.edu.gh)

**Table S1. Reported therapeutic coverage of ivermectin Mass Drug Administration in the study communities (2009–2010)**

| **Study locations** | **2009**  **(1st round)** | | | **2009**  **(2nd round)** | | | **2010**  **(1st round)** | | |
| --- | --- | --- | --- | --- | --- | --- | --- | --- | --- |
|  | **Treated/**  **Total popn.**  **(%)** | **Non-eligibles**  **(%)** | **Absent & Refused**  **(%)** | **Treated/**  **Total popn.**  **(%)** | **Non-eligibles**  **(%)** | **Absent & Refused**  **(%)** | **Treated/**  **Total popn.**  **(%)** | **Non-eligibles**  **(%)** | **Absent & Refused**  **(%)** |
| **Tain District** |  |  |  |  |  |  |  |  |  |
| Abekwai 2 | 125/143 (87.4%) | 10/143  (7.0%) | 8/143  (5.6%) | 118/135 (87.4%) | 15/135  (11.1%) | 2/135  (1.5%) | 106/121 (87.6%) | 15/121  (12.4%) | 0/121  (0%) |
| Abekwai 3 | 391/489 (80.0%) | 70/489  (14.3%) | 28/489  (5.7%) | 290/372 (78.0%) | 54/372  (14.5%) | 28/372  (7.5%) | 328/394 (83.3%) | 58/394  (14.7%) | 8/394  (2.0%) |
| Attakrom | 128/177 (72.3%) | 49/177  (27.7%) | 0/177  (0.0%) | 188/208  (61.5%) | 14/208  (6.7%) | 6/208  (2.9%) | 198/231 (85.7%) | 14/231  (6.1%) | 19/231  (8.2%) |
| Kokomba | ‒ | ‒ | ‒ | ‒ | ‒ | ‒ | ‒ | ‒ | ‒ |
| **Total Tain** | **644/809**  **(79.6%)** | **129/809**  **(16.0%)** | **36/809**  **(4.5%)** | **596/715**  **(83.4%)** | **83/715**  **(11.6%)** | **36/715**  **(5.0%)** | **632/746**  **(84.7%)** | **87/746**  **(11.7%)** | **27/746**  **(3.6%)** |
| **Wenchi Municipality** |  |  |  |  |  |  |  |  |  |
| Blibor | ‒ | ‒ | ‒ | ‒ | ‒ | ‒ | ‒ | ‒ | ‒ |
| Johnykrom | ‒ | ‒ | ‒ | ‒ | ‒ | ‒ | ‒ | ‒ | ‒ |
| Subinso 1 | 657/790 (83.2%) | 131/790  (16.6%) | 2/790  (0.3%) | ‒ | ‒ | ‒ | ‒ | ‒ | ‒ |
| Subinso 2 | ‒ | ‒ | ‒ | ‒ | ‒ | ‒ | ‒ | ‒ | ‒ |
| Kwanware | 53/64  (82.8%) | 10/64  (15.6%) | 1/64  (1.6%) | ‒ | ‒ | ‒ | ‒ | ‒ | ‒ |
| **Total Wenchi** | **710/854**  **(83.1%)** | **141/854**  **(16.5%)** | **3/854**  **(0.5%)** | ‒ | ‒ | ‒ | ‒ | ‒ | ‒ |

**Table S2. Reported therapeutic coverage of ivermectin Mass Drug Administration in the study communities (2019–2021)**

| **Study locations** | **2019**  **(1st round)** | | | **2019**  **(2nd round)** | | | **2021**  **(1st round)** | | | **2021**  **(2nd round)** | | |
| --- | --- | --- | --- | --- | --- | --- | --- | --- | --- | --- | --- | --- |
|  | **Treated/**  **Total popn.**  **(%)** | **Non-eligibles**  **(%)** | **Absent & Refused**  **(%)** | **Treated/**  **Total popn.**  **(%)** | **Non-eligible**  **(%)** | **Absent & Refused**  **(%)** | **Treated/**  **Total popn.**  **(%)** | **Non-eligibles**  **(%)** | **Absent & Refused**  **(%)** | **Treated/**  **Total popn.**  **(%)** | **Non-eligibles**  **(%)** | **Absent & Refused**  **(%)** |
| **Tain District** |  |  |  |  |  |  |  |  |  |  |  |  |
| Abekwai 2 | 390/476  (81.9%) | 71/476  (14.9%) | 15/476  (3.2%) | 395/476  (83.0%) | 64/476  (13.5%) | 17/476  (3.6%) | 390/476  (81.9%) | 78/476  (16.4%) | 8/476  (1.7%) | 405/476  (85.1%) | 63/476  (13.2%) | 8/476  (1.7%) |
| Abekwai 3 | 802/971  (82.6% | 155/971  (16.0%) | 14/971  (1.4%) | 803/971  (82.7%) | 155/971  (16.0%) | 13/971  (1.3%) | 802/971  (82.6%) | 150/971  (15.5%) | 19/971  (2.0%) | 808/971  (83.2%) | 144/971  (14.8%) | 19/971  (2.0%) |
| Attakrom | 417/515  (81.0%) | 88/515  (17.0%) | 10/515  (1.9%) | 417/515  (81.0%) | 98/515  (19.0%) | 0/515  (0%) | 416/520  (80.0%) | 72/520  (13.9%) | 32/520  (6.2%) | 422/520  (81.2%) | 92/520  (17.7%) | 6/520  (1.2%) |
| Kokomba | 400/495  (80.8%) | 88/495  (17.8%) | 7/495  (1.4%) | 404/495  (80.8%) | 85/495  (17.2%) | 6/495  (1.2%) | 399/495  (80.6%) | 76/495  (15.4%) | 20/495  (4.0%) | 408/495  (82.4%) | 81/495  (16.4%) | 6/495  (1.2%) |
| **Total Tain** | **2,009/2,457**  **(81.8%)** | **402/2,457**  **(16.4%)** | **46/2,457**  **(1.9%)** | **2,019/2,457**  **(82.2%)** | **402/2,457**  **(16.4%)** | **36/2,457**  **(1.5%)** | **2,007/2,462**  **(81.5%)** | **376/2,462**  **(15.3%)** | **79/2,462**  **(3.2%)** | **2,043/2,462**  **(83.0%)** | **380/2,462**  **(15.4%)** | **39/2,462**  **(1.6%)** |
| **Wenchi Municipality** |  |  |  |  |  |  |  |  |  |  |  |  |
| Blibor | ‒ | ‒ | ‒ | 187/240  (77.9%)* | 39/240  (16.3%) | 14/240  (5.8%) | 244/304  (80.3%)* | 50/304  (16.5%) | 10/304  (3.3%) | ‒ | ‒ | ‒ |
| Johnykrom | ‒ | ‒ | ‒ | ‒ | ‒ | ‒ | ‒ | ‒ | ‒ | ‒ | ‒ | ‒ |
| Subinso 1 |  |  |  | 853/1,036  (82.3%)* | 171/1,036  (16.5%) | 12/1,036  (1.2%) | 883/1,080  (81.8%)* | 178/1,080  (16.5%) | 19/1,080  (1.8%) | ‒ | ‒ | ‒ |
| Subinso 2 | ‒ | ‒ | ‒ | 1,972/2,451  (80.5%)* | 404/2,451  (16.5%) | 75/2,451  (3.1%) | ‒ | ‒ | ‒ | ‒ | ‒ | ‒ |
| Kwanware | ‒ | ‒ | ‒ | 78/99  (78.8%)* | 16/99  (16.2%) | 5/99  (5.1%) | 76/96  (79.2%)* | 16/96  (16.7%) | 4/96  (4.2%) | ‒ | ‒ | ‒ |
| **Total Wenchi** | ‒ | ‒ | ‒ | **3,090/3,826**  **(80.8%)*** | **630/3,826**  **(16.5%)** | **106/3,826**  **(2.8%)** | **1,203/1,480**  **(81.3%)*** | **244/1,480**  **(16.5%)** | **33/1,480**  **(2.2%)** | ‒ | ‒ | ‒ |

* Adjusted according to the proportion of non-eligible population in Wenchi Municipality.

**Figure S1.** Temporal trends of microfilarial prevalence for Abekwai (Tain District)

and Kwanware (Wenchi Municipality)


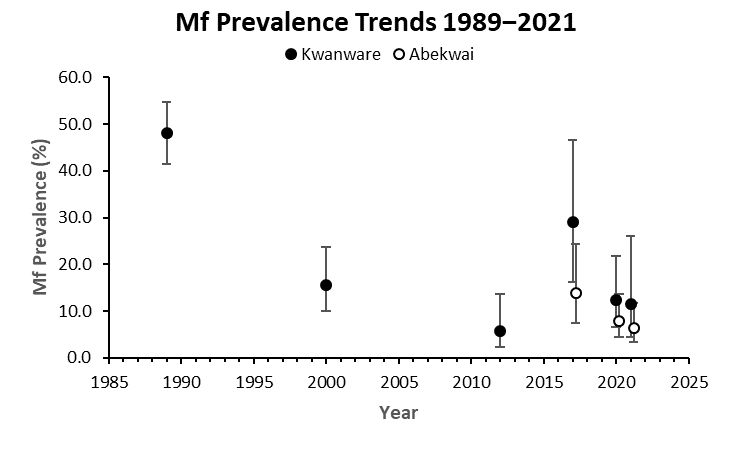


The value of mf prevalence for Kwanware in 1989 represents the pre-control (baseline) value from the OCP database. The value for 2000 corresponds to the last epidemiological evaluation before the closure of the OCP. The value of 2012 is from the ESPEN portal for Ghana and corresponds to an impact assessment evaluation funded by the APOC Trust Fund.

For Abekwai, the mf prevalence in 2020 and 2021 was calculated by combining the data for Abekwai 2 and Abekwai 3, to be able to compare with the 2017 data point for Abekwai. The values for 2017 are from an impact assessment evaluation conducted by GHS.

There was no CDTI in 2020 and therefore the data for 2020 correspond to one year after the last treatment round in 2019. The data for 2021 were collected five months after the first treatment round in 2021.

Error bars are the Wilson interval 95% confidence intervals.

See Main text for Abbreviations used.
